# Supplementary material for: Culturing of a complex gut microbial community in mucin-hydrogel carriers reveals strain- and gene-associated spatial organization
Source: Nat Commun. 2023 Jun 14;14:3510. doi: 10.1038/s41467-023-39121-0 (PMC10267222; doi:10.1038/s41467-023-39121-0)
Supplement: Supplementary file 1 — Supplementary Information [file 41467_2023_39121_MOESM1_ESM.pdf]

Supplementary Information

Hybrid Nanopore Illumina genome assembly approach

As described in the Methods – Hybrid assembly of microbial isolates, we use a hybrid assembly approach combining Nanopore and Illumina reads from isolate strain DNA to generate closed genomes for each strain in the community. Each strain is cultured in its preferred medium in an anaerobic environment until it reaches stationary phase to recover cell pellets. In order to preserve the length of genomic DNA during extraction, cell pellets are subjected to more gentle bead beating (10 Hz for 5 min) and enzymatic lysis (Lucigen). Because columns tend to shear genomic DNA during the extraction process, high molecular weight DNA is isolated from cell lysate using phenol chloroform approaches and pelleted using ethanol precipitation. We resuspend DNA in elution buffer (Qiagen) and quantify both length and concentration of the extracted DNA (Agilent, ThermoFisher). We try to achieve a length of $>$ 10 kbp at $>$ 200 ng per strain.

To generate both long and short read data, we use MinION (Oxford Nanopore) and NovaSeq (Illumina). Oxford Nanopore libraries are generated using the PCR-free ligation kit (LSK109). Because long read sequencing can be cost prohibitive at scale, we multiplex 4-8 strains on the same MinION flow cell using ligation barcoding expansion kits (NBD104, NBD114), yielding 200k reads with N50 of 6-9 kbp. With bacterial genome sizes of 5 Mbp, 200k Nanopore reads cover the genome more than 100X. To alleviate the increased error rate of Nanopore long reads, we supplement Nanopore long reads for each strain with 2-3 million Illumina read pairs, which achieves a short read coverage of 100X and can be obtained at $10 inclusive of library preparation. Together, our pipeline to acquire 100X fold coverage of both long Nanopore reads and short Illumina reads for all 123 strains require a cost of $200 per strain, inclusive of reagents and consumables of all experimental steps.

Hybrid assembly of strain genomes is carried out using a custom bioinformatic approach built around Unicycler (Fig. S1) [1]. Unicycler can handle as input data a maximum of 100X coverage for long reads and short reads separately. Therefore, long reads are first filtered and sub-sampled for quality and length, whereas short reads are filtered by quality and then coverage normalized. Unicycler takes as input filtered reads and, if possible, creates a closed and polished assembly. When the assembly is not closed, scaffolding using LRScaf [2] and gap closing using TGS-GapCloser [3] are performed in an attempt to generate a complete reference genome. The strain hybrid assembly pipeline using Nanopore and Illumina reads is available at

`https://github.com/FischbachLab/fischbachlab/nf-hybridassembly`.

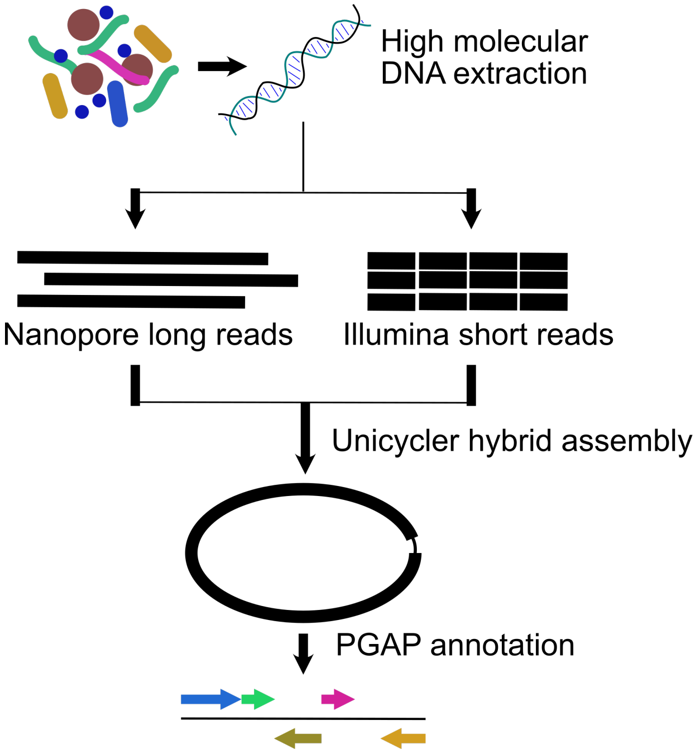

Figure S1: Schematic of hybrid assembly approach.

Synthetic community strains

Table S1 lists information for all 123 strains in our community. This community is closely modeled after hCom1 and hCom2 from Cheng *et al.* 2021 [4]. We include all strains from both hCom1 and hCom2 in order to start with a comprehensive community. We also include several additional strains (*Peptostreptococcus anaerobius* ATCC 27337, *Peptostreptococcus sp.* CC14N HM 1051, *Clostridium sp.* D5, *Turicibacter sanguinis* DSM 14220) that are known to produce important metabolites *in vivo* [5–7]. In addition, we exclude *Bacteroides rodentium* DSM 26882 because it is not a strain isolated from the human gut, as well as *Lactobacillus plantarum ATCC-BAA-793*. Media and inoculation order describe growth of strains as isolates prior to community assembly. Slow growing fastidious strains are inoculated first 3 days prior to community assembly, intermediate strains are inoculated second 2 days prior to assembly, and fastest growing strains are inoculated third 1 day prior to assembly. Taxonomic classification obtained by running de novo genomes through GTDB-tk [8]. We manually curate closest available NCBI genomes using strain identifier keyword search, prioritizing complete genomes when multiple entries are present. Closest ANI matches to representative species in UHGG database [9] are determined using FastANI [10], which was also used to determine ANI with closest available NCBI genome. Kofamscan [11] was used to obtain KO mappings for both de novo and closest NCBI genome, differential KOs (diffKOs) are counted as those for which the maximum hit bitscore in the de novo genome is more than 2-fold different from the NCBI genome.

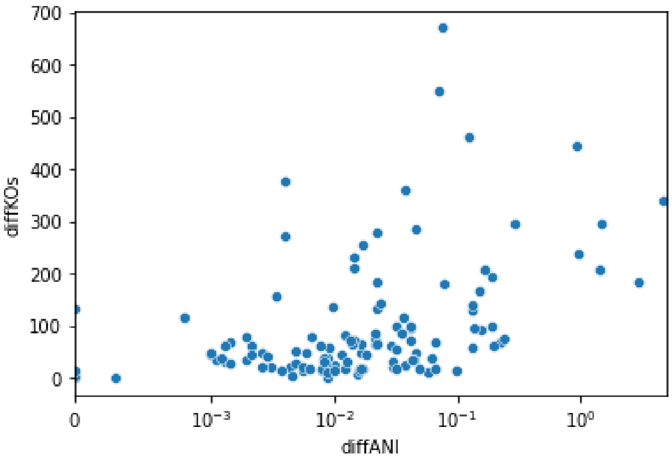

Figure S2: diffANI (100%-ANI) plotted against diffKO plotted for all 123 community strains, comparing de novo vs. NCBI closest genomes.

Anaerobic chamber and liquid handling setup

To enable large scale liquid handling required for fast and error-free community assembly from isolate strain cultures, we build a custom anaerobic chamber infrastructure that includes liquid handling capabilities in close proximity to culture incubator space and plate reader.

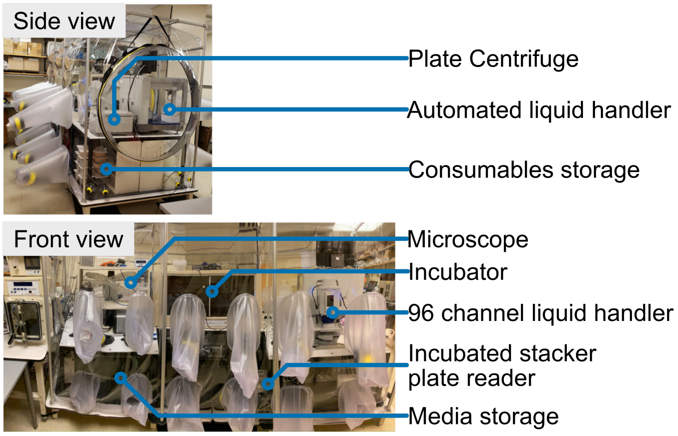

Figure S3: Labeled picture of anaerobic chamber system used for experiments.

DNA extraction and metagenomic sequencing libraries

A total of 276 DNA samples are extracted (2 sampling $\times$ 3 sequencing replicates for inoculum, plus 6 passages $\times$ 5 experimental conditions (mucin agar carriers, mucin agar supernatant, plain agar carriers, plain agar supernatant, no-carrier control) $\times$ 3 biological replicates (separate culture tubes, i.e., R1, R2, R3) $\times$ 3 technical replicates

(within culture tubes, e.g., R1a, R1b, R1c). Across samples, we measure mean DNA concentration $7.3ng/uL$ – read libraries generated from these samples after library prep and shotgun metagenomic sequencing exhibit mean read depth 1.2×10^7. Table S2 details abundance (read fraction) and horizontal coverage for each strain for each read library. Table S3 details log carrier enrichment scores from paired carrier-vs.-supernatant samples, as well as aggregated carrier enrichment scores for both plain-agar and mucin-agar carrier cultures.

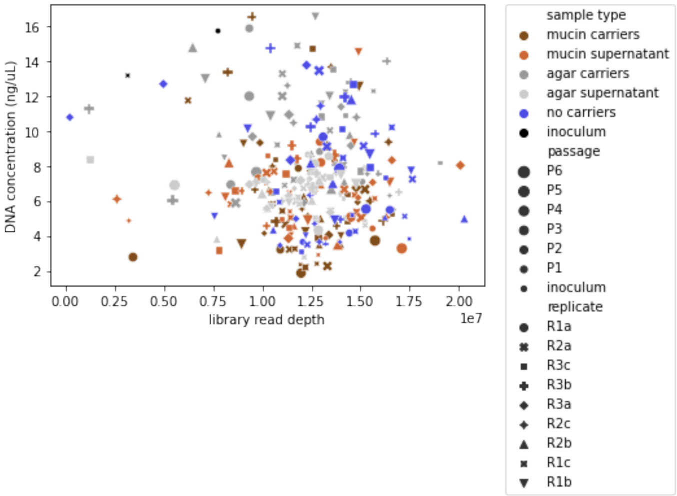

Figure S4: DNA concentration plotted against library read depth for each sample.

Comparing abundance quantification using Ninjamap with custom database against Kraken2+Bracken with UHGG database and custom database

To validate the read abundances determined using Ninjamap with our custom strain database, we use the same read libraries and quantify read abundance using Kraken2 [12]+Bracken [13] with an existing gut species database – UHGG [9]. For each strain, we compare the relative abundance calculated by NinjaMap with that of the closest UHGG species determined by Kraken2+Bracken. Note that for several strains, there is not a 1-1 correspondence between strain and UHGG species as multiple strains all have the same closest UHGG species (maximum strain correspondence up to 4). In these cases, we sum the NinjaMap relative abundances of all corresponding strains prior to comparing against Kraken2+Bracken estimated UHGG species abundance. Across all passage / experimental condition / replicate samples (270 total), we find strong correlation between NinjaMap and Kraken2+Bracken relative abundances (median R^2 of 0.987760). By contrast, if we compare Ninjamap results with Kraken2+Bracken using a custom database built from our community strain genomes, correlation improves even further to median R^2 of 0.999442. Like Kraken2+Bracken, Ninjamap reports read-fraction as final output - while this fraction is not adjusted for genome size, the algorithm does use genome size in determining the proportion of escrowed reads to assign to each strain.

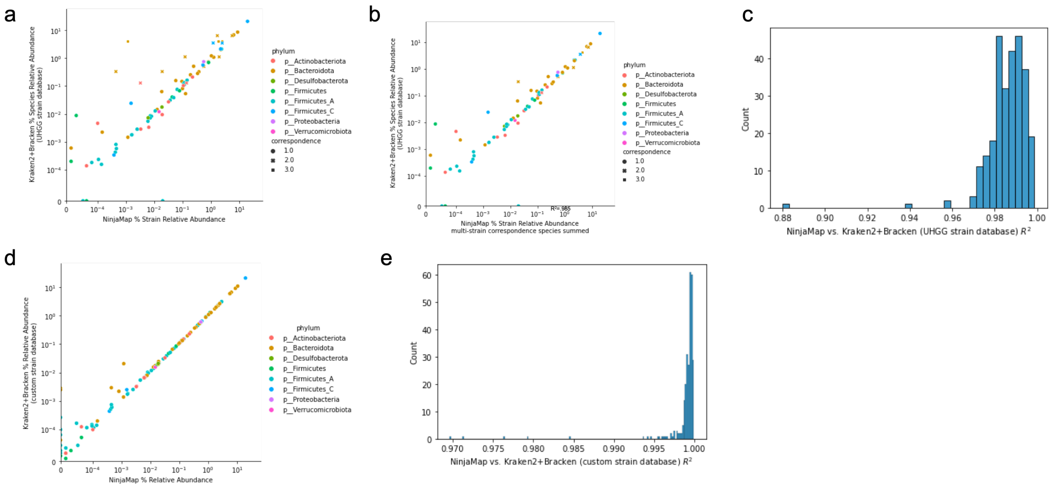

Figure S5: Comparison between relative abundances determined by NinjaMap with custom de novo genomes database versus Kraken2+Bracken with UHGG database. **a:** Scatterplot of NinjaMap vs. Kraken2+Bracken relative abundances for each strain, mapped to closest UHGG species, using the no-carrier control passage 1 replicate 1a sample library. Most strains have a 1-1 correspondence to closest UHGG species, but certain species have up to 3 corresponding strains. **b:** Scatterplot of NinjaMap vs. Kraken2+Bracken relative abundances for each strain, mapped to closest UHGG species, summing NinjaMap abundances in cases where species have multiple corresponding strains. **c:** Distribution of R^2 values for NinjaMap vs. Kraken2+Bracken (UHGG database) relative abundances across all passage / experimental condition / replicate samples (270 total). **d:** Scatterplot of NinjaMap vs. Kraken2+Bracken relative abundances with Kraken2 using custom database built from community strain genomes instead of the UHGG database employed in A-C. **e:** Distribution of R^2 values for NinjaMap vs. Kraken2+Bracken with the custom strain database, including relative abundances across all passage / experimental condition / replicate samples (270 total). Correlations are even higher than in C.

Community richness subset by phylum, including plain-agar carriers

Counting detected strains (1% horizontal coverage and 0.0001% relative abundance cutoff) subset by phylum, we demonstrate that most of diversity gain in our synthetic community due to carrier addition occurs in Bacteroides, Firmicutes_A, and Firmicutes. We also plot counts for plain-agar carrier / supernatant samples, showing that this abundance increase occurs when plain-agar carriers are added, though to a lesser extent than with mucin-agar carriers. Finally we compare strain counts (i.e. community richness) from late passage (P3-P6) no-carrier, mucin carrier, mucin supernatant samples. In addition to these 3 experimental conditions we also include a 4th pseudo-condition – mucin readsmix – where NinjaMap analysis is done using pooled mucin carrier and mucin supernatant reads (each downsampled at 50% to adjust for total read number) from the same culture tube.

Regardless of how mucin carrier cultures are sampled (on carriers, in supernatant, or pseudomix of reads from both), these cultures exhibit significantly more detected strains (i.e., community richness) than no-carrier cultures. Considering only final passage P6, and collapsing technical replicates into their median value for each biological

replicate (i.e., n=3), one-way ANOVA comparison between four conditions yields overall $p = 1.4 \times 10^{-6}$, with pairwise post-hoc Tukey HSD $p = 8.4 \times 10^{-6}, 2.5 \times 10^{-6}, 3.3 \times 10^{-6}$ between no-carriers versus mucin carriers, mucin supernatant, and mucin readsmix respectively. Pairwise post-hoc Tukey HSD $p > 0.05$ between all mucin conditions.

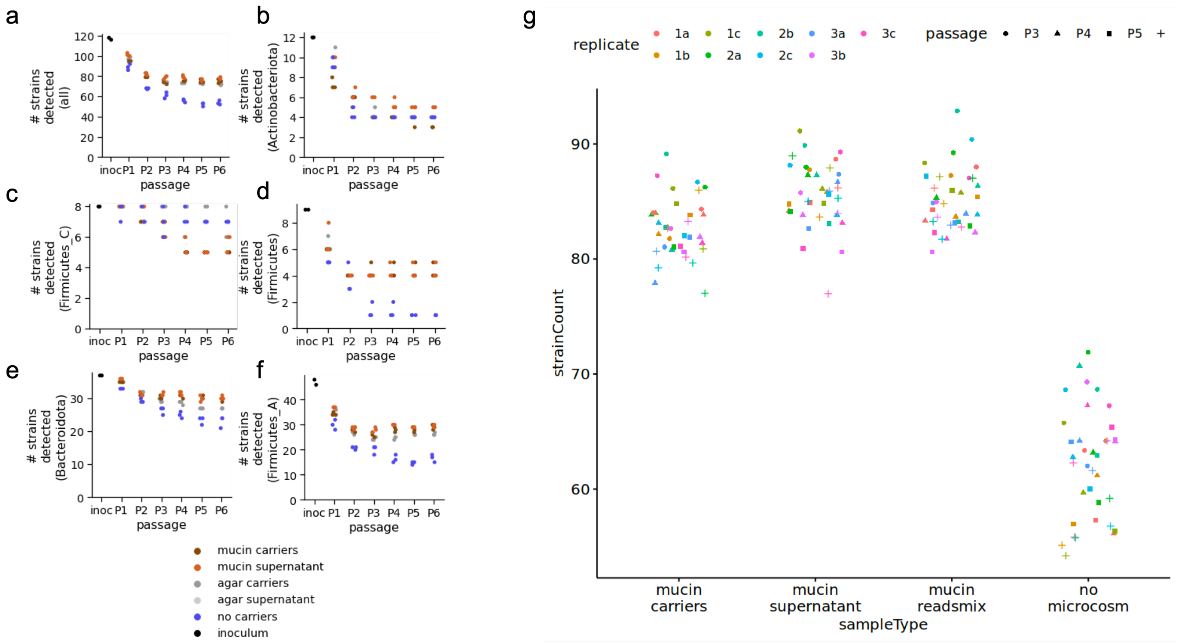

Figure S6: Counts of strains detected (1% horizontal coverage and 0.0001% relative abundance cutoff) across passages, subset by phylum, including data from mucin-agar carrier/supernatant, plain-agar carrier/supernatant, and no-carrier samples. **a:** Considering all phyla, strain counts are higher when carriers are present and slightly lower with plain-agar carriers versus mucin-agar carriers. **b:** Actinobacteria does not exhibit strong diversity gain with addition of carriers. **c:** Firmicutes_C (~Negativicutes) does not exhibit strong diversity gain with addition of carriers. **d:** Firmicutes (~Bacillus) exhibits strong diversity gain with addition of carriers. **e:** Bacteroidota exhibits strong diversity gain with addition of carriers. **f:** Firmicutes_A (~Clostridia) exhibits strong diversity gain with addition of carriers, particularly mucin-agar. **g:** Comparing all late-passage data points (passages P3-P6, biological replicates 1-3, technical replicates a-c, total n=36) for mucin carrier, mucin supernatant, mucin readsmix and no carrier conditions. Strain counts in no-carrier cultures are significantly lower than all mucin conditions.

Comprehensive heatmap of abundance measurements including plain agar carrier cultures and additional non-prevalent strains

We plot here additional heat maps and graphs that include abundance data from the experiment, including data for plain agar carrier cultures (carrier and supernatant), for the full 123 community (including less prevalent strains). We also provide additional examples of abundance patterns between related strains.

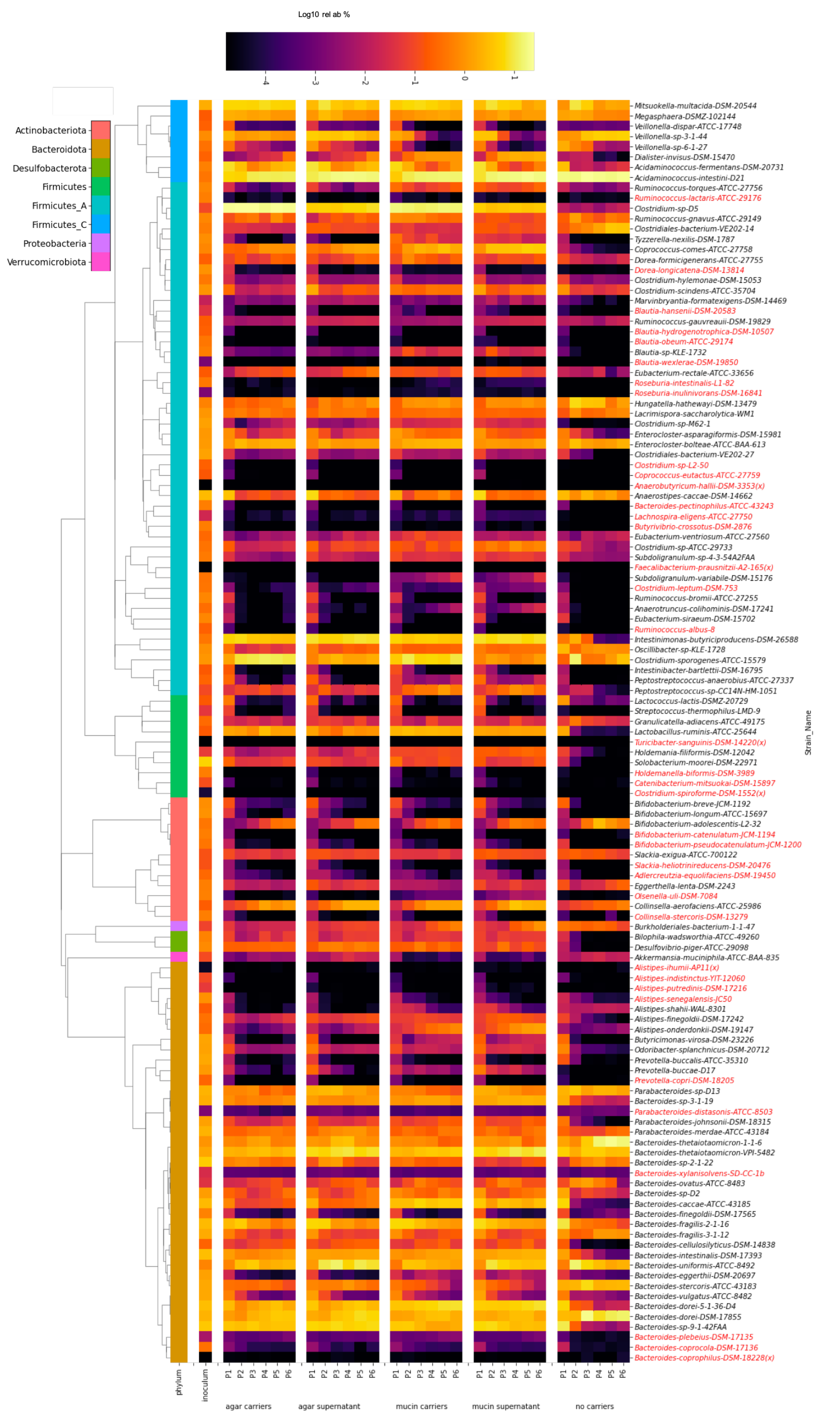

Figure S7: All abundances from across all 5 experimental conditions (mucin agar carrier/supernatant, plain agar carrier/supernatant, no carrier control) and 6 passages plotted as heatmap, taking median of 3 biological replicates, which are themselves median of 3 technical replicates. Strains highlighted in red were not in the top 86 top prevalent strains and were not included in the phylogenetic regression analysis described in the main text. Strains with low abundance ($< 10^{-4}\%$) in inoculum are those which failed to grow from glycerol stock isolates, marked with (x).

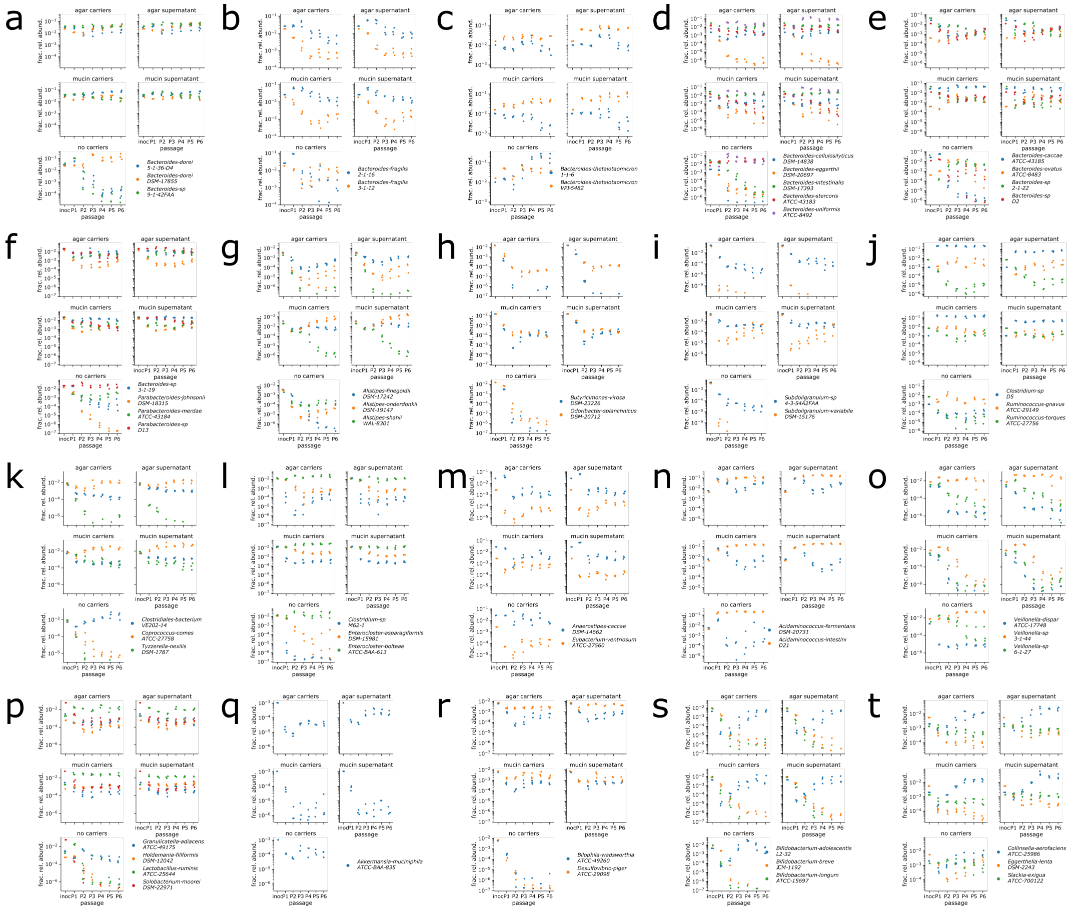

Figure S8: **(Previous page)** Selected abundance comparisons between related strains, across all 5 experimental conditions (mucin agar carrier/supernatant, plain agar carrier/supernatant, no carrier control) and 6 passages. 3 biological replicates points plotted, each point represents median of 3 technical replicates. **a:** 3 *B. dorei* strains show coexistence with carriers, and coexistence occurs with both plain-agar and mucin-agar carriers (extension of Fig. 1F) **g:** 2 *B. fragilis* strains show increased abundance of one (2-1-16) relative to the other (3-1-12) upon addition of carriers. **c:** 2 *B. thetaiotaomicron* strains show one strain (1-1-6) with reduced abundance while the other (*VPI-5482*) is unaffected upon addition of carriers. **d:** 5 Bacteroides strains show greater coexistence with carriers, with *B. cellulosilyticus* and *B. intestinalis* in particular exhibiting higher abundance with carriers. *B. eggerthii* exhibits this to a lesser extent, only with mucin-agar carriers, which appears to coincide with decreased abundance of *B. stercoris*. *B. uniformis* remains highly abundant in all conditions. **e:** 4 additional Bacteroides strains show coexistence with carriers, with known mucin forager *B. caccae* in particular exhibiting higher abundance with carriers. **f:** 4 Parabacteroides strains show coexistence with carriers, with *P. johnsonii* in particular exhibiting higher abundance with carriers. **g:** 3 Alistipes strains show higher abundance with carriers of *A. finegoldii* and *A. onderkii* (particularly mucin-agar). These increases appear to come at the expense of *A. shahii*, which is the most abundant Alistipes strain without carriers, but least abundant with carriers. **h:** 2 Odoribacter strains show neither grow well without carriers – growth is rescued for both in the presence of mucin-agar carriers, but in plain-agar carriers culture, only *O. splanchnicus* is rescued. **i:** 2 Subdoligranulum strains show coexistence with mucin-agar carriers, but not plain-agar carriers (extension of Fig. 1G) **j:** 3 Lachnospiraceae strains show distinct responses to presence of carriers: sp. D5 exhibits improved growth in the presence of either mucin agar or plain agar carriers, *R. torques* only for mucin agar carriers, while *R. gnavus* does not exhibit large abundance changes to carrier presence. **k:** 3 additional Lachnospiraceae strains shows coexistence with carriers. Only strain *VE202-14* is abundant without carriers, but *C. comes* in particular comes more abundant with mucin-agar and plain-agar carriers added, while *T. nexilis* growth is rescued with mucin-agar carriers only. **l:** 3 additional Lachnospiraceae strains shows coexistence with carriers, while *E. bolteae* dominates among the three without carriers **m:** 3 additional Lachnospiraceae strains shows approximately similar abundances with and without carriers. **n:** 2 Acidaminococcus strains show coexistence with carriers, and coexistence occurs with both plain-agar and mucin-agar carriers (extension of Fig. 1H) **o:** 3 Veillonella strains show reduced abundance upon addition of mucin agar carriers, particularly for *sp. 3-1-44* – this effect is less pronounced for plain-agar carriers. **p:** 4 Bacillus strains shows higher abundances with carriers, particularly for *L. ruminis*. **q:** *Akkermansia muciniphila ATCC-BAA-835* show lower abundance when mucin-agar carriers are present, compared with both plain-agar carriers and no-carrier control. **r:** 2 Desulfobacter strains show both have higher growth when carriers are present. **s:** 3 Bifidobacterium strains show patterns which are not strongly affected by carriers (*B. adolescentis* dominates in all conditions). **t:** 3 Coriobacteria strains show distinct responses to presence of carriers: *C. aerofaciens* exhibits improved growth in the presence of carriers, *E. lenta* exhibits reduced growth, while *S. exigua* appears relatively unaffected by comparison.

Principal component analysis to visualize community strain composition across sample types and timepoints

We apply principal component analysis (PCA) to the strain abundance vectors for all samples to visually compare relationships in communities across different sample types (mucin-agar vs. plain-agar, supernatant vs. carrier vs. no-carrier-control) and passages. We apply standard scaling prior to PCA, and we visualize using the top two principal components. We find that considering all samples including the inoculum, the first primary component is dominated by differences between the inoculum samples and all passage samples. By running this analysis on the passage samples only, and especially using only the late passage samples (P3-6), we find that datapoints cluster repeatably by sample type, with differences between passage being most prominent in early passages (P1 is most distinct) and becoming more modest by P3-6. The observation that replicates from each sample type cluster together indicates that clear and repeatable differences exist in community composition between (i) cultures with carriers and those without carriers, (ii) cultures with mucin added to the hydrogel carriers vs. plain-agar, (iii) samples from the carrier fraction vs. the supernatant fraction of the same culture, both for plain-agar and mucin-agar carriers.

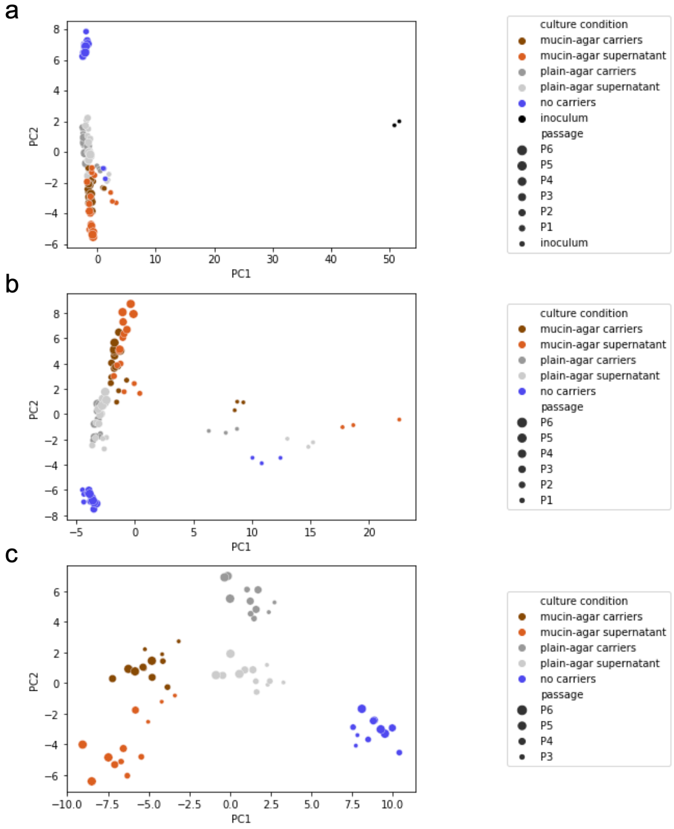

Figure S9: Principal component analysis (PCA) visualization of community composition across sample types and passages. **a:** Across all samples, the primary differences in the first 2 principal components visualized (PC1/PC2) exist between inoculum and passaged samples, which cluster far apart – this aligns with higher community richness in the inoculum. 3 replicates each for passaged timepoints, 2 replicates for inoculum – abundance matrix is standard-scaled (mean and standard deviation normalized to 0 and 1 respectively for each strain) prior to PCA. **b:** Analyzing only passaged samples, PCA visualization indicates differences between sample types, with plain-agar vs. mucin-agar and carrier vs. supernatant vs. no-carrier samples clustering separately. We also observe that the first (and to a much lesser extent second) passages P1-P2 also cluster away from the later passage samples – however, even by passage 1, different conditions begin to differentiate. **c:** Analyzing only late passage samples (P3 onward), we observe clear and repeatable differences between sample types, with more modest effects based on passage number, aligning with overall stability observed in community richness from passage P3 onward.

Carrier enrichment with plain agar carriers

Here we show carrier enrichment calculations with the plain agar carriers, both as heatmap of individual strain as well as aggregated by phylum. We also highlight some additional enrichment patterns between strains. Comparing plain-agar enrichment with mucin-agar enrichment we find that enrichment on plain-agar carriers is generally a good predictor of enrichment on mucin-agar carriers, as seen based on positive correlation between two metrics across strains. These comparisons enable us to disentangle the effects of adding mucin-hydrogel surface from simply adding a hydrogel surface into the culture environment, and thus allow us to test whether the simple presence of mucin on the hydrogel surface impacts community organization. Indeed, for a subset of strains, we find greater than expected mucin-agar carrier enrichment than would be predicted based solely on their plain-agar carrier enrichment including known mucin foragers *Akkermansia muciniphila* and *Bacteroides caccae*.

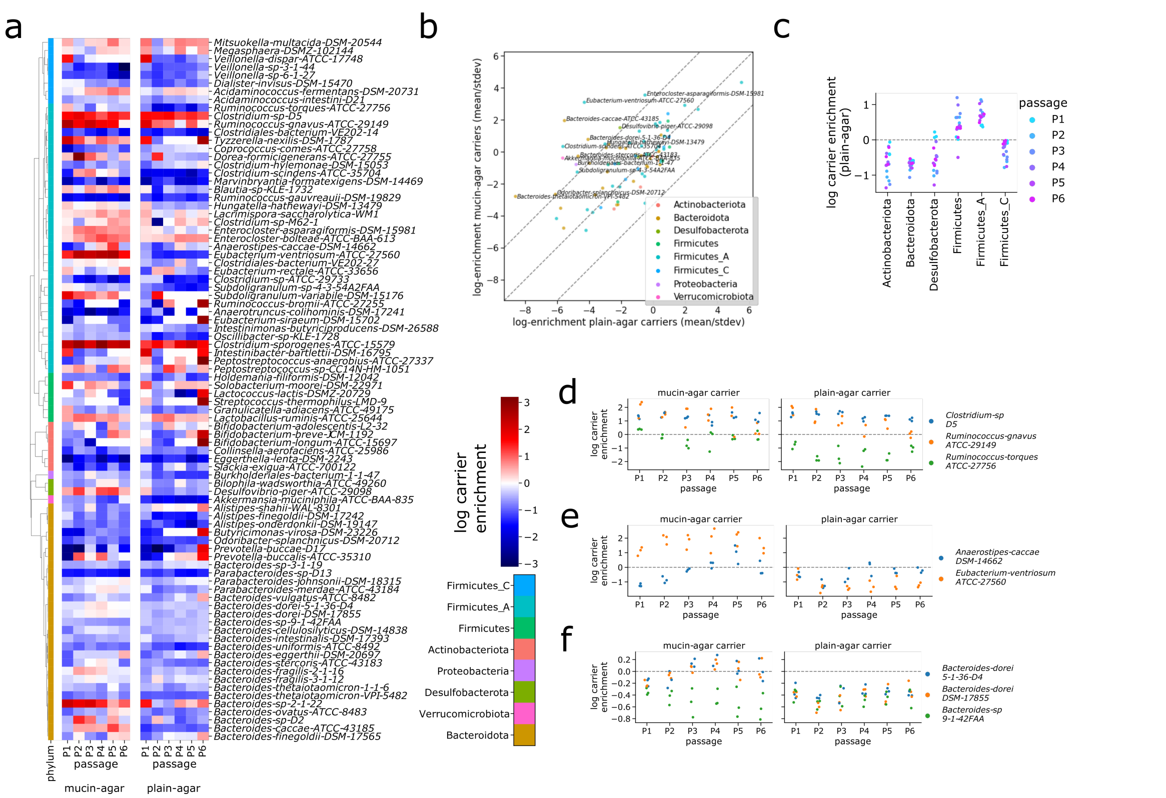

Figure S10: Comparison between carrier enrichment with mucin-agar and plain-agar carriers. **a:** Heatmap of mucin-agar (left, copied from Fig. 2A) and plain-agar carrier enrichment scores – positive (red) scores indicate carrier enrichment. **b:** Scatterplot of mucin-agar enrichment vs. plain-agar enrichment across 86 top prevalent strains. Position of each point represents mean over standard deviation of enrichment scores across 12 late passage replicates (4 late passages x 3 replicates = 12 total samples). Positive correlation (Two-sided Pearson's product-moment correlation test $R^2 = 0.55$, $p = 4.9 \times 10^{-08}$) indicates that strains enriched on plain-agar carriers also tend to be enriched on mucin-agar carriers – dashed lines indicate equality and 2 SD bounds. Several strains with greater than expected mucin-agar enrichment based on their plain-agar enrichment are highlighted, including known mucin foragers *Akkermansia muciniphila* and *Bacteroides caccae*. **c:** Aggregated at phylum level, results are similar in plain-agar carrier cultures as mucin-agar with the exception of Desulfobacterota, which is now enriched in supernatant (compare with Fig. 2B). **d:** *Clostridium sp. D5* exhibits carrier enrichment for both mucin-agar and plain-agar. **e:** *Eubacterium ventriosum* exhibits carrier enrichment for mucin-agar only. **f:** All three strains of *B. dorei* exhibit carrier depletion in plain-agar case.

Growth rate estimates using peak-trough coverage ratio analysis

To explore whether differences may be due to different growth rates between culture conditions (e.g., carrier-attached vs. liquid supernatant) for certain strains, we apply the peak-to-trough coverage ratio method on our metagenomic read libraries to estimate growth rates across strains, timepoints and culture conditions – this was done with the iRep software package, using the bPTR implementation to take advantage of the availability of closed genomes.

Across all samples, we find a median of 35 strains per sample have read sufficient coverage for pBTR to generate a PTR growth rate estimate. We find that for these strains, most PTR values are close to 1. This is consistent with expectations that the the culture has more or less reached stationary phase by the time of sampling (3 days / passage), though there are some exceptions such as *Mitsuokella multicida DSM 20544* and *Lactobacillus ruminis ATCC 25644* which consistently exhibit ratios higher than 1, indicative of active growth at time of sampling.

We next compare PTR scores between carrier-attached vs. liquid supernatant culture conditions, by taking the ratio of PTR scores generated from mucin-agar carrier samples by PTR scores generated from the corresponding supernatant samples. Across all strains for which we are able to generate PTR estimates, we fail to observe any instances where this carrier-attached vs. liquid supernatant culture PTR ratio is significantly different from 1, meaning there are no consistent differences in PTR values between cultures sampled from the mucin carriers compared with the surrounding liquid phase for any strains. This suggests that observed mucin carrier strain enrichment / depletion cannot be fully attributed to growth rate differences between conditions. As a caveat, these samples were taken at the end of 3-day passages, and therefore it is somewhat expected that many strains have likely settled toward stationary phase – it is possible that future experiments with higher sampling frequencies may yield growth rate differences across strains and conditions by capturing cultures that are in the exponential growth phase.

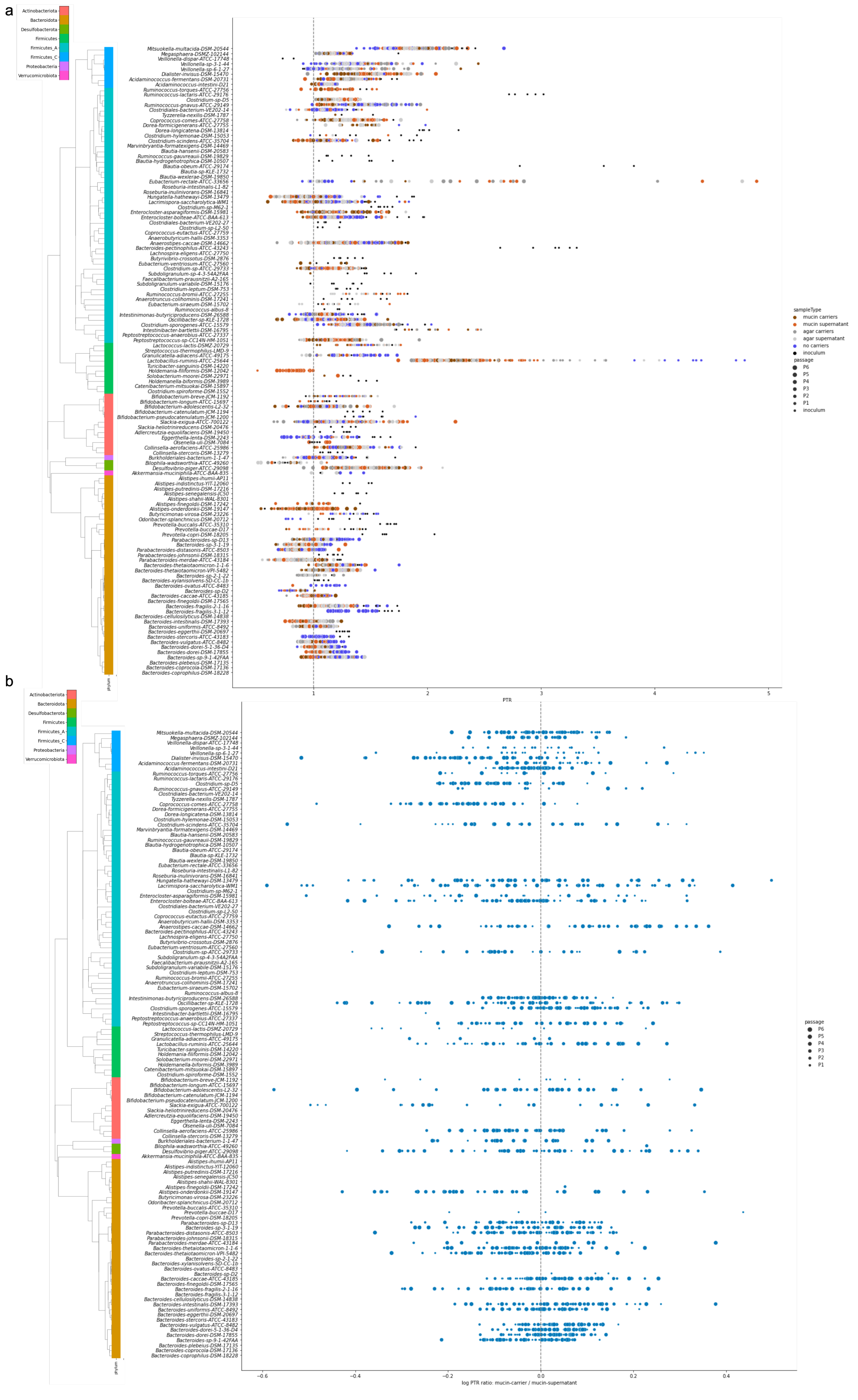
a
Actinobacteriota
Bacteroidota
Desulfobacterota
Firmicutes
Firmicutes_A
Firmicutes_C
Proteobacteria
Verrucomicrobiota
phylum
sampleType
mucin carriers
mucin supernatant
agar carriers
agar supernatant
no carriers
inoculum
passage
P6
P5
P4
P3
P2
P1
inoculum
PTR
b
log PTR ratio: mucin-carrier / mucin-supernatant

Figure S11: **(Previous page)** Peak-to-trough ratios for most strains are around unity, suggesting limited active growth by end of each passage – no clear differences in PTR growth estimates between mucin carrier and supernatant samples for any strains. **a:** Peak-to-trough ratios (PTR) for strains, colored by culture condition – most values are around 1, suggesting the culture has more or less reached stationary phase by the time of sampling (3 days / passage), though there are some exceptions such as Mitsuokella multicida and Lactobacilllus ruminis which have higher PTR values indicative of more active growth. **b:** Comparing PTR growth estimates by taking the ratio of PTR between corresponding mucin-carrier and mucin-supernatant samples, we do not observe any strains with carrier-vs.-supernatant PTR ratios that deviate significantly from 1, indicating similar growth rates on the carriers vs. in the supernatant.

Gene difference comparisons between strains

We search for differential KO / gene family presence between strain genomes by applying a 1.5-fold hmmer bitscore cutoff for each KO. For example, if a strain has a maximum bitscore of 10 for a particular KO, any strain with a maximum bitscore greater than 15 or less than 6.67 will be considered to have a differential KO presence. Table S4 lists maximum bitscore hits for K00441 and K08217 KO pHMMs against Bacteroides dorei DSM-17855 / Bacteroides dorei 5-1-36-D4 / Bacteroides sp. 9-1-42FAA, as well as the maximum bitscore hits for K14440 in Subdoligranulum variabile-DSM-15176 / Subdoligranulum-sp-4-3-54A2FAA, and K14743 in Acidaminococcus fermentans DSM-20731 / Acidaminococcus intestini D21, supporting the examples from Fig. 2C-E.

Gene neighbourhood analysis of K00441

Table S5 lists frequency of co-occurrence between every KO and K00441 (number of instances they exist within 10kb of each other, across all community genomes), as well as the number of times out of 1000 random permutations (all gene labels shuffled across all genomes) that the actual frequency exceeds the random permutation.

In vivo dataset analysis

We use the Suez *et al.* 2018 dataset [14] as a source of metagenomic read libraries from *in vivo* gut microbial samples, with paired lumen and mucosa samples within individuals, at multiple gastrointestinal tract locations. We initially select 16 individuals with lumen and mucosa data from terminal ileum, cecum and ascending colon. We then use Kneaddata (part of Biobakery suite []) to filter reads based on quality and to remove human (host) reads. After this filtering step, 13/16 individuals remain, corresponding to a total of 78 read libraries – Table S6 details read libraries used.

We then use Kraken2 [12] to classify filtered reads from each of these libraries against the UHGG database [9], obtaining phyla-level and species-level relative abundance estimates for each read library. We focus our analysis

on species that are detected with at least 0.01% abundance in at least 10% of libraries, leading to a subset of 676 species. For each of these species, we calculate their mucosal enrichment score by comparing paired mucosa/lumen samples from the same individual and site, applying the log-ratio approach as done for carrier enrichment with the in-vitro dataset. We additionally generate an aggregate score by taking the mean over standard deviation of log-ratios. Aggregate scores are generated both per individual (over 3 sites), as well as across all individuals to generate a single aggregate score per species. These enrichment scores are detailed in Table S7. We also calculated phyla-level enrichment scores for each paired lumen/mucosa sample, plotted in Fig. S12A. Similar trends exist between *in vivo* and *in vitro* enrichments at phylum level: Bacteroidota is enriched toward both supernatant (*in vitro*) and lumen (*in vivo*), while Firmicutes_A (Clostridia-like) and Firmicutes (Bacillus-like) are enriched toward carrier / mucosa. However, discrepancies also exist, as Actinobacteriota is enriched toward supernatant *in vitro* and mucosa *in vivo*.

For the species-level results, we compare the Spearman correlation of *in vivo* mucosal enrichment scores across species with *in vitro* carrier enrichment scores (both mucin-agar and plain agar) across strains, mapping *in vitro* strains to their closest UHGG species and only considering taxa that pass the prevalence threshold (0.01% abundance in at least 10% of samples) in both analyses. We find significant positive pairwise Spearman correlation scores between *in vivo* mucosal-enrichment and *in vitro* carrier-enrichment scores using plain agar carriers ($p<0.001$), as well as *in vitro* plain-agar carriers and mucin carriers scores ($p<0.001$). Correlation is positive but not significant ($p=0.16$) between *in vivo* and *in vitro* mucin carriers. Stratifying the *in vivo* dataset by human participants, we observe significant variability between participants, who group into two main clusters. Our *in vitro* log-carrier-enrichment scores (plain-agar and mucin-agar carriers) group within the larger of these two clusters, indicating that observed discrepancy between *in vitro* and *in vivo* mucosal/carrier scores does not exceed inter-subject variability.

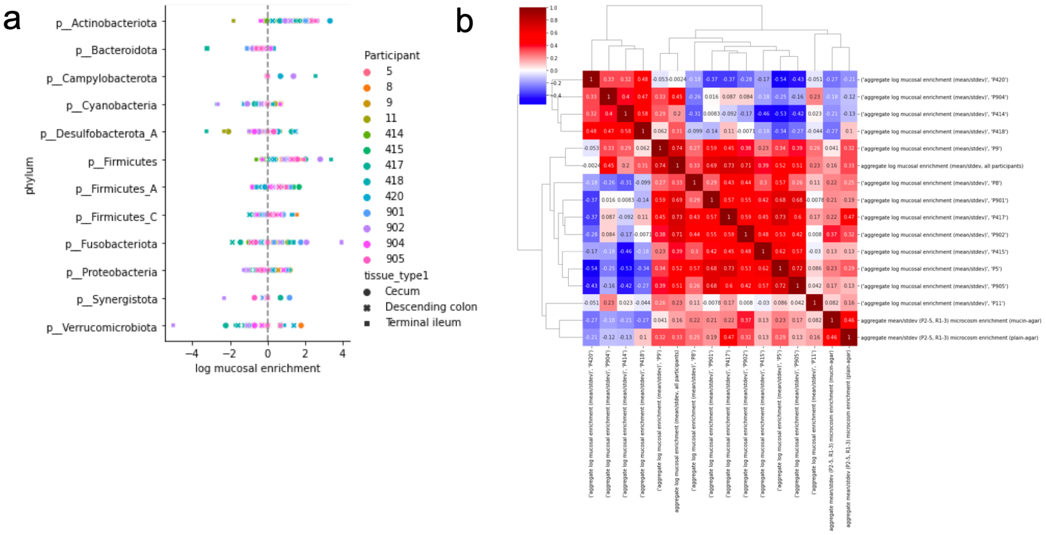

Figure S12: Mucosal enrichments from *in vivo* data [14]. **a:** Mucosal enrichments from *in vivo* dataset at phylum level. **b:** Spearman correlation of mucosal enrichments at species-level, compared with *in vitro* strain carrier enrichmments.

Detailed results from phylogenetic linear models

Table S8 lists all phylogenetic linear model results for all tested KOs. For the 244 carrier-associated KOs (i.e., effect size > 0) that pass Benjamini-Hochberg FDR significance test at FDR<0.01. We also show clade specific results in Table S9, where only the subset of strains belonging to a single clade are considered when performing the the phylogenetic linear model – note FDR requirement is relaxed for clade specific significance testing to increase sensitivity. We group phylum Firmicutes, Firmicutes_A and Firmicutes_C into a single clade for this analysis. The majority of significant phyla-specific hits (217) occur within Bacteroidota – which has numerous instances of closely related strains – followed by Firmicutes (5); other phyla with fewer strain representatives did not produce clade-specific significant hits. We also include results from parallel analysis using the Suez 2018 *in vivo* dataset (Table S10), where we identify a total of 6831 significant KO hits associated with increased mucosal enrichment. Comparing the *in vitro* and *in vivo* KO hits (Fig. S13), we find that 199 KOs are associated with both increased mucosal (*in vivo* dataset [14]) and carrier (*in vitro* dataset from our own work) enrichment, 45 that are only carrier-enrichment associated, 6632 that are only mucosal-enrichment associated, and 5984 that are neither, corresponding to a $log-odds-ratio = 3.99, 95\%CI2.89-5.65, p < 2.2 \times 10^{-16}$, using a two-sided Fisher's exact test – confirming significant overlap between *in vivo* and *in vitro* results.

In vivo data (Suez 2018)		In vitro data (this work): Significant microcosm enriched	In vitro data (this work): Not significant microcosm enriched
	Significant mucosa enriched	199	45
	Not significant mucosa enriched	6632	5984

Figure S13: 2x2 contingency table showing overlap between significant KO hits for carrier enrichment (*In vitro* data from this work) and significant KO hits for mucosa enrichment (*In vivo* data from Suez 2018 [14]). Log odds ratio of 3.99 and $p < 1^{-20}$ using a 2x2 fisher exact test.

Aggregative KEGG BRITE analysis

For each KEGG BRITE hierarchical category, we consider all KOs that fall under the category's umbrella. We then intersect this subset of BRITE KO's with the 244 significantly carrier-associated KOs identified earlier, to generate a 2x2 contingency table (carrier-associated KOs in BRITE category, non-carrier-associated KOs in BRITE category, carrier-associated KOs not in BRITE category, and non-carrier-associated KOs not in BRITE category). We then use this table to calculate log odds ratio p values using a fisher exact test – this yields a total of 43 BRITE categories significantly $(p < 0.05)$ associated with increased carrier enrichment, listed in Table S11.

Identifying and grouping biosynthetic gene clusters using DeepBGC and hierarchical clustering

We use DeepBGC [15] to identify BGCs in our genomes, yielding a total of 1349 BGCs in our community genomes. For each BGC, we determine KEGG KO presence by considering all CDSs within the BGC – all KO's mapped to any CDS within the BGC (at least 0.5x hmmer bitscore relative to the KEGG-defined bitscore threshold, and 0.5x length overlap relative to total pHMM length) are considered present in the BGC. Based on this we focus on 1103 BGCs with at least 3 mapped KOs (and conversely KOs with at least 3 mapped BGCs), yielding a boolean 1103 BGC x 1387 KO presence absence matrix. We apply hierarchical clustering on this matrix using Jaccard distance metric to generate 256 groups of BGCs (see Table S12), and subsequently map each BGC back to its original strain. This finally yields a boolean 86 strain x 256 BGC-group presence absence matrix (again focusing on the 86 top prevalent strains, see Fig. S14). We then test iteratively using phylolm [16] one BGC-group at a time for significant association with carrier enrichment across strains. Note that there are instances where numerous BGC-groups exhibit very similar genotypes across these 86 top prevalent strains, leading to the case where a number of BGC-groups exhibit similar p-values (see Fig. 5B). Table S13 lists KOs in the resulting 7 significantly carrier-enriched BGC-groups.

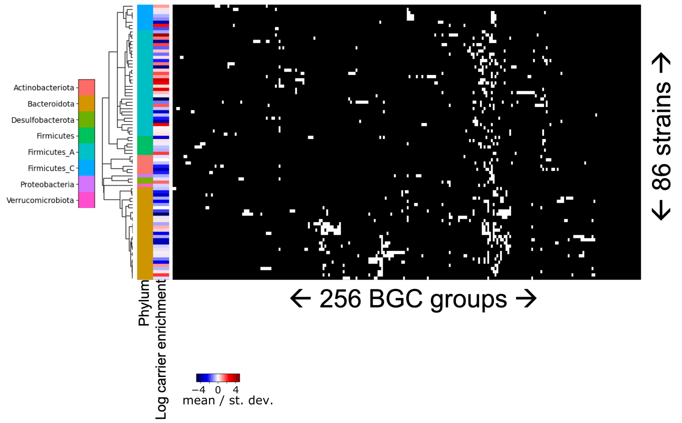

Figure S14: BGC-group by strain presence/absence matrix, focusing on 86 top prevalent strains. White indicates BGC-group is present in strain, black indicates absence.

References

[1] Ryan R Wick, Louise M Judd, Claire L Gorrie, and Kathryn E Holt. Unicycler: Resolving bacterial genome assemblies from short and long sequencing reads. *PLOS Computational Biology*, 13(6):e1005595, jun 2017.

[2] Mao Qin, Shigang Wu, Alun Li, Fengli Zhao, Hu Feng, Lulu Ding, and Jue Ruan. LRScaf: improving draft genomes using long noisy reads. *BMC genomics*, 20(1):1–12, 2019.

[3] Mengyang Xu, Lidong Guo, Shengqiang Gu, Ou Wang, Rui Zhang, Brock A Peters, Guangyi Fan, Xin Liu, Xun Xu, and Li Deng. TGS-GapCloser: a fast and accurate gap closer for large genomes with low coverage of error-prone long reads. *GigaScience*, 9(9):giaa094, 2020.

[4] Alice G Cheng, Andrés Aranda-Díaz, Sunit Jain, Feiqiao Yu, Mikhail Iakiviak, Xiandong Meng, Allison Weakley, Advait Patil, Anthony L Shiver, Adam Deutschbauer, Norma Neff, Kerwyn Casey Huang, and Michael A Fischbach. Systematic dissection of a complex gut bacterial community. *bioRxiv*, page 2021.06.15.448618, jan 2021.

[5] Jonathan B Lynch, Erika L Gonzalez, Kayli Choy, Kym F Faull, Talia Jewell, Abelardo Arellano, Jennifer Liang, Kristie B Yu, Jorge Paramo, and Elaine Y Hsiao. Turicibacter modifies host bile acids and lipids in a strain-specific manner. *bioRxiv*, page 2022.06.27.497673, jan 2022.

[6] Julia H Kemis, Vanessa Linke, Kelsey L Barrett, Frederick J Boehm, Lindsay L Traeger, Mark P Keller, Mary E Rabaglia, Kathryn L Schueler, Donald S Stapleton, Daniel M Gatti, Gary A Churchill, Daniel Amador-Noguez, Jason D Russell, Brian S Yandell, Karl W Broman, Joshua J Coon, Alan D Attie, and Federico E Rey. Genetic determinants of gut microbiota composition and bile acid profiles in mice. *PLOS Genetics*, 15(8):e1008073, aug 2019.

[7] Marta Wlodarska, Chengwei Luo, Raivo Kolde, Eva D'Hennezel, John W Annand, Cortney E Heim, Philipp Krastel, Esther K Schmitt, Abdifatah S Omar, Elizabeth A Creasey, Ashley L Garner, Sina Mohammadi, Daniel J O'Connell, Sahar Abubucker, Timothy D Arthur, Eric A Franzosa, Curtis Huttenhower, Leon O Murphy, Henry J Haiser, Hera Vlamakis, Jeffrey A Porter, and Ramnik J Xavier. Indoleacrylic Acid Produced by Commensal Peptostreptococcus Species Suppresses Inflammation. *Cell Host and Microbe*, 22(1):25–37.e6, 2017.

[8] Pierre-Alain Chaumeil, Aaron J Mussig, Philip Hugenholtz, and Donovan H Parks. GTDB-Tk: a toolkit to classify genomes with the Genome Taxonomy Database. *Bioinformatics*, 36(6):1925–1927, mar 2020.

[9] Alexandre Almeida, Stephen Nayfach, Miguel Boland, Francesco Strozzi, Martin Beracochea, Zhou Jason Shi, Katherine S Pollard, Ekaterina Sakharova, Donovan H Parks, Philip Hugenholtz, Nicola Segata, Nikos C Kyrpides, and Robert D Finn. A unified catalog of 204,938 reference genomes from the human gut microbiome. *Nature Biotechnology*, 39(1):105–114, 2021.

[10] Chirag Jain, Luis M Rodriguez-R, Adam M Phillippy, Konstantinos T Konstantinidis, and Srinivas Aluru. High throughput ANI analysis of 90K prokaryotic genomes reveals clear species boundaries. *Nature Communications*, 9(1):5114, 2018.

[11] Takuya Aramaki, Romain Blanc-Mathieu, Hisashi Endo, Koichi Ohkubo, Minoru Kanehisa, Susumu Goto, and Hiroyuki Ogata. KofamKOALA: KEGG Ortholog assignment based on profile HMM and adaptive score threshold. *Bioinformatics*, 36(7):2251–2252, apr 2020.

[12] Derrick E Wood, Jennifer Lu, and Ben Langmead. Improved metagenomic analysis with Kraken 2. *Genome Biology*, 20(1):257, 2019.

[13] Jennifer Lu, Florian P Breitwieser, Peter Thielen, and Steven L Salzberg. Bracken: estimating species abundance in metagenomics data. *PeerJ Computer Science*, 3:e104, 2017.

[14] Jotham Suez, Zamir Halpern, Eran Segal, and Eran Elinav. Personalized Gut Mucosal Colonization Resistance to Empiric Probiotics Is Associated with Unique Host and Microbiome Features Article Personalized Gut Mucosal Colonization Resistance to Empiric Probiotics Is Associated with Unique Host and Microbiome Feat. *Cell*, 174(6):1388–1405.e21, 2018.

[15] Geoffrey D Hannigan, David Prihoda, Andrej Palicka, Jindrich Soukup, Ondrej Klempir, Lena Rampula, Jindrich Durcak, Michael Wurst, Jakub Kotowski, Dan Chang, Rurun Wang, Grazia Piizzi, Gergely Temesi, Daria J Hazuda, Christopher H Woelk, and Danny A Bitton. A deep learning genome-mining strategy for biosynthetic gene cluster prediction. *Nucleic Acids Research*, 47(18):e110–e110, oct 2019.

[16] Lam Si Tung Ho and Cecile Ane. A linear-time algorithm for Gaussian and non-Gaussian trait evolution models. *Systematic Biology*, 63:397–408, 2014.
