# Supplementary material for: Culturing of a complex gut microbial community in mucin-hydrogel carriers reveals strain- and gene-associated spatial organization
Source: Nat Commun. 2023 Jun 14;14:3510. doi: 10.1038/s41467-023-39121-0 (PMC10267222; doi:10.1038/s41467-023-39121-0)
Supplement: Supplementary file 3 — Description of Additional Supplementary Files [file 41467_2023_39121_MOESM3_ESM.docx]

File Name: SupplementaryDataS1.csv

Description: Strain metadata information for 123 strains, including BioProject accessions and BioSample IDs. Genomes from strains with BioProject accessions PRJNA746600 and PRJNA885826 were generated for this study, other genomes were obtained from NCBI. Note that this table also contains information for 2 additional strains that were excluded from our experiments (L. plantarum and B. rodentium).

File Name: SupplementaryDataS2.csv

Description: Strain abundances for each strain and each sample, generated using NinjaMap.

File Name: SupplementaryDataS3.csv

Description: Strain abundance log-enrichments (carrier over supernatant) for paired carrier/supernatant samples, as well as aggregated over all late passage P3-P6 samples for both mucin-agar and plain-agar conditions. We also include calculations of log-enrichment of mucin-agar carrier over plain-agar carrier.

File Name: SupplementaryDataS4.csv

Description: Kofamscan bitscores (i.e., similarity to KEGG Orthology database entry) for each gene in genome database

File Name: SupplementaryDataS5.csv

Description: Genomic colocalization of KOs relative to K00441 genes in our database

File Name: SupplementaryDataS6.csv

Description: Metadata for read libraries used from Suez / Zmora et al 2018 (PMIDs 30193112 and 30193113) in vivo biopsy dataset with paired lumen/mucosal samples.

File Name: SupplementaryDataS7.csv

Description: Species abundance log-enrichments (mucosa over lumen) calculated from paired mucosa/lumen samples, from Suez / Zmora et al 2018.

File Name: SupplementaryDataS8.csv

Description: KO associations with increased mucin-carrier/mucin-supernatant enrichment scores, calculated using phylogenetic linear regression model using Brownian Motion model for covariance (implemented using phylolm R package) - reported p values are two-sided. Associations are computed across top 86 prevalent strains, and include FDR adjusted p-values using Benjamini/Hochberg.

File Name: SupplementaryDataS9.csv

Description: KO associations with increased mucin-carrier/mucin-supernatant enrichment scores, subsetting by phylum.

File Name: SupplementaryDataS10.csv

Description: KO associations with increased mucosa/lumen enrichment scores, calculated using species abundance log-enrichments calculated from Suez / Zmora et al 2018, using same statistical approach as Table S8.

File Name: SupplementaryDataS11.csv

Description: Enumeration and significance testing (two-sided Fisher exact test) of KEGG BRITE categories based on the number of significant KO hits associated with increased mucin-carrier/mucin-supernatant enrichment.

File Name: SupplementaryDataS12.csv

Description: Table of biosynthetic gene clusters (BGCs) detected using deepbgc, including their label information after grouping into BGC groups based on KOs detected in each BGC.

File Name: SupplementaryDataS13.csv

Description: Most prevalent KOs in 7 BGC groups (groups 157, 120, 198, 34, 186, 69, and 161) associated with increased carrier/supernatant enrichment.

File Name: SupplementaryDataS14.csv

Description: KO associations with increased agar-carrier/agar-supernatant enrichment scores, calculated across top 86 prevalent strains. (Equivalent to Table S8 but for plain-agar instead of mucin-agar carrier cultures)

File Name: SupplementaryDataS15.csv

Description: Enumeration and significance testing of KEGG BRITE categories based on the number of significant KO hits associated with increased agar-carrier/agar-supernatant enrichment. (Equivalent to Table S11 but for plain-agar instead of mucin-agar carrier cultures)

File Name: SupplementaryDataS16.csv

Description: Metadata on read libraries for metagenomes, available from NCBI SRA under BioProject PRJNA885585. Note that passage numbers in raw filenames are 0-indexed, while final manuscript uses 1-indexing to avoid confusion with inoculum. E.g., CZBMI-Biofilm_BeadExperiment_ExpV2_AB_P0R1a_position_M13 corresponds to data from plain agar carrier sample, passage P1 in the manuscript.
