# Supplementary material for: Culturing of a complex gut microbial community in mucin-hydrogel carriers reveals strain- and gene-associated spatial organization
Source: Nat Commun. 2023 Jun 14;14:3510. doi: 10.1038/s41467-023-39121-0 (PMC10267222; doi:10.1038/s41467-023-39121-0)
Supplement: Supplementary file 5 — Reporting Summary [file 41467_2023_39121_MOESM5_ESM.pdf]

nature portfolio

Corresponding author(s): Katherine S. Pollard

Last updated by author(s): May 15, 2023

Reporting Summary

Nature Portfolio wishes to improve the reproducibility of the work that we publish. This form provides structure for consistency and transparency in reporting. For further information on Nature Portfolio policies, see our Editorial Policies and the Editorial Policy Checklist.

Statistics

For all statistical analyses, confirm that the following items are present in the figure legend, table legend, main text, or Methods section.

n/a	Confirmed	
☐	☒	The exact sample size (*n*) for each experimental group/condition, given as a discrete number and unit of measurement
☐	☒	A statement on whether measurements were taken from distinct samples or whether the same sample was measured repeatedly
☐	☒	The statistical test(s) used AND whether they are one- or two-sided<br>*Only common tests should be described solely by name; describe more complex techniques in the Methods section.*
☐	☒	A description of all covariates tested
☐	☒	A description of any assumptions or corrections, such as tests of normality and adjustment for multiple comparisons
☐	☒	A full description of the statistical parameters including central tendency (e.g. means) or other basic estimates (e.g. regression coefficient) AND variation (e.g. standard deviation) or associated estimates of uncertainty (e.g. confidence intervals)
☐	☒	For null hypothesis testing, the test statistic (e.g. *F*, *t*, *r*) with confidence intervals, effect sizes, degrees of freedom and *P* value noted<br>*Give P values as exact values whenever suitable.*
☒	☐	For Bayesian analysis, information on the choice of priors and Markov chain Monte Carlo settings
☒	☐	For hierarchical and complex designs, identification of the appropriate level for tests and full reporting of outcomes
☐	☒	Estimates of effect sizes (e.g. Cohen's *d*, Pearson's *r*), indicating how they were calculated

Our web collection on statistics for biologists contains articles on many of the points above.

Software and code

Policy information about availability of computer code

Data collection	No software was used
Data analysis	Software used for this project include:<br>constructing and annotating genomes: Unicycler 0.4.8, LRScaf 1.1.9, TGS-GapCloser 1.1.1, PGAP 2021-01-11.build5132<br>Microbial community analysis: ninjamap 1.1.0, gtdb-tk 1.5.0 kofamscan 1.3.0, deepbgc 0.1.27, phylolm 2.6.2, fastani 1.32, scipy 1.5.3, pandas 1.1.4, numpy 1.19.2, seaborn 0.11.0, python 3.7.10, ipython 5.8.0, jupyter notebook 6.1.4, statsmodels 0.12.1, dna_features_viewer 3.1.0, biopython 1.78, reportlab 3.5.68, matplotlib 3.3.2, irep 1.1.14, R 4.1.3, ggtree v3.2.1, treeio v1.18.1, ggnewscale 0.4.7, phytools_1.0-3, RColorBrewer_1.1-3, tidyr_1.2.1, dplyr_1.0.10, stringr_1.4.1, ggplot2_3.3.6, cowplot_1.1.1, ape_5.6-2<br>Custom code used for the project available at:<br>https://github.com/xiaofanjin/gut-community-microcosms<br>https://github.com/FischbachLab/nf-hybridassembly

For manuscripts utilizing custom algorithms or software that are central to the research but not yet described in published literature, software must be made available to editors and reviewers. We strongly encourage code deposition in a community repository (e.g. GitHub). See the Nature Portfolio guidelines for submitting code & software for further information.

Data

Policy information about availability of data

All manuscripts must include a data availability statement. This statement should provide the following information, where applicable:

- Accession codes, unique identifiers, or web links for publicly available datasets
- A description of any restrictions on data availability
- For clinical datasets or third party data, please ensure that the statement adheres to our policy

GTDB [https://gtdb.ecogenomic.org/], UHGG [https://www.ebi.ac.uk/metagenomics], and KEGG KO [https://www.genome.jp/kegg/ko.html] databases are publicly available. Metagenomic sequencing data is deposited under NCBI Bioproject PRJNA885585 [https://www.ncbi.nlm.nih.gov/search/all/?term=PRJNA885585] while hybrid genomes (along with underlying illumina / nanopore sequencing reads) are deposited under PRJNA885826 [https://www.ncbi.nlm.nih.gov/search/all/?term=PRJNA885826] and to BioProject PRJNA746600 [https://www.ncbi.nlm.nih.gov/search/all/?term=PRJNA746600]. Details for each strain are listed in Table S1 including BioProject accession number as well as individual BioSample IDs. Full source data used for analysis is available at Figshare dataset gut-community-microcosms [https://figshare.com/articles/dataset/gut-community-microcosms/21094717.]

Human research participants

Policy information about studies involving human research participants and Sex and Gender in Research.

Reporting on sex and gender	This study did not involve human research participants
Population characteristics	This study did not involve human research participants
Recruitment	This study did not involve human research participants
Ethics oversight	This study did not involve human research participants

Note that full information on the approval of the study protocol must also be provided in the manuscript.

Field-specific reporting

Please select the one below that is the best fit for your research. If you are not sure, read the appropriate sections before making your selection.

- [x] Life sciences
- [ ] Behavioural & social sciences
- [ ] Ecological, evolutionary & environmental sciences

For a reference copy of the document with all sections, see nature.com/documents/nr-reporting-summary-flat.pdf

Life sciences study design

All studies must disclose on these points even when the disclosure is negative.

Sample size	No sample size calculations were performed for in vitro experiments, which were performed with biological triplicate and technical triplicate (i.e., 9 total samples for each condition) as detailed in the text. Triplicates were chosen to enable replication statistics, while maintaining a feasible scale for the total experiment. Similar levels of variation are observed between biological and technical replicates. No other experiments applicable.
Data exclusions	No data was excluded
Replication	Enhanced community richness, and coexistence with microcosm cultures for highlighted (B. dorei, Subdoligranulum and Acidaminococcus in main text, additional examples in supplements) strains were reproducible across replicates, as were differential microcosm enrichment across strains from these taxa. Replication done in triplicate, all attempts at replication successful. No other experiments applicable.
Randomization	Randomization not applicable as in vitro cultures were inoculated from a common inoculum community. No other experiments applicable.
Blinding	Complete blinding not possible due to visually obvious differences while sampling from microcosm, supernatant and liquid cultures; all sampling was performed by a single investigator. No other experiments applicable.

Reporting for specific materials, systems and methods

We require information from authors about some types of materials, experimental systems and methods used in many studies. Here, indicate whether each material, system or method listed is relevant to your study. If you are not sure if a list item applies to your research, read the appropriate section before selecting a response.

Materials & experimental systems

n/a	Involved in the study
☒	☐ Antibodies
☒	☐ Eukaryotic cell lines
☒	☐ Palaeontology and archaeology
☒	☐ Animals and other organisms
☒	☐ Clinical data
☒	☐ Dual use research of concern

Methods

n/a	Involved in the study
☒	☐ ChIP-seq
☒	☐ Flow cytometry
☒	☐ MRI-based neuroimaging
